# The Brief Case: Wound infection following a penetrating and cutting instrument injury: a case report

**DOI:** 10.1128/jcm.01472-25

**Published:** 2026-07-08

**Authors:** Mustafa Yilmaz, Ayşen Zeynep Dik, Fatma Erdem, Simay Erşahin, Yakup Karabağli

**Affiliations:** 1 Faculty of Medicine, Department of Medical Microbiology, Eskisehir Osmangazi University 53004, Eskişehir, Turkey; 2 Faculty of Medicine, Department of Plastic, Reconstructive, and Aesthetic Surgery, Eskisehir Osmangazi University, Eskişehir, Turkey; Endeavor Health, Evanston, Illinois, USA

**Keywords:** animal contact, MALDI-TOF MS, wound infection, *Trueperella pyogenes*, livestock exposure

## CASE

A 35-year-old male presented to the emergency department after sustaining a deep hand injury while slaughtering cattle with a saw. Lacerations and fractures of the first, second, and fifth fingers of the right hand were surgically repaired. Because of penicillin allergy, prophylaxis was started with ciprofloxacin and gentamicin; however, due to allergic symptoms, ciprofloxacin was replaced by clindamycin on postoperative day 3. C-reactive protein increased from 32.8 to 87.2 mg/L in the first 3 days. On day 5, redness and purulent discharge developed in the right second finger, and aspirated fluid was sent for microbiological examination.

**Fig 1:**
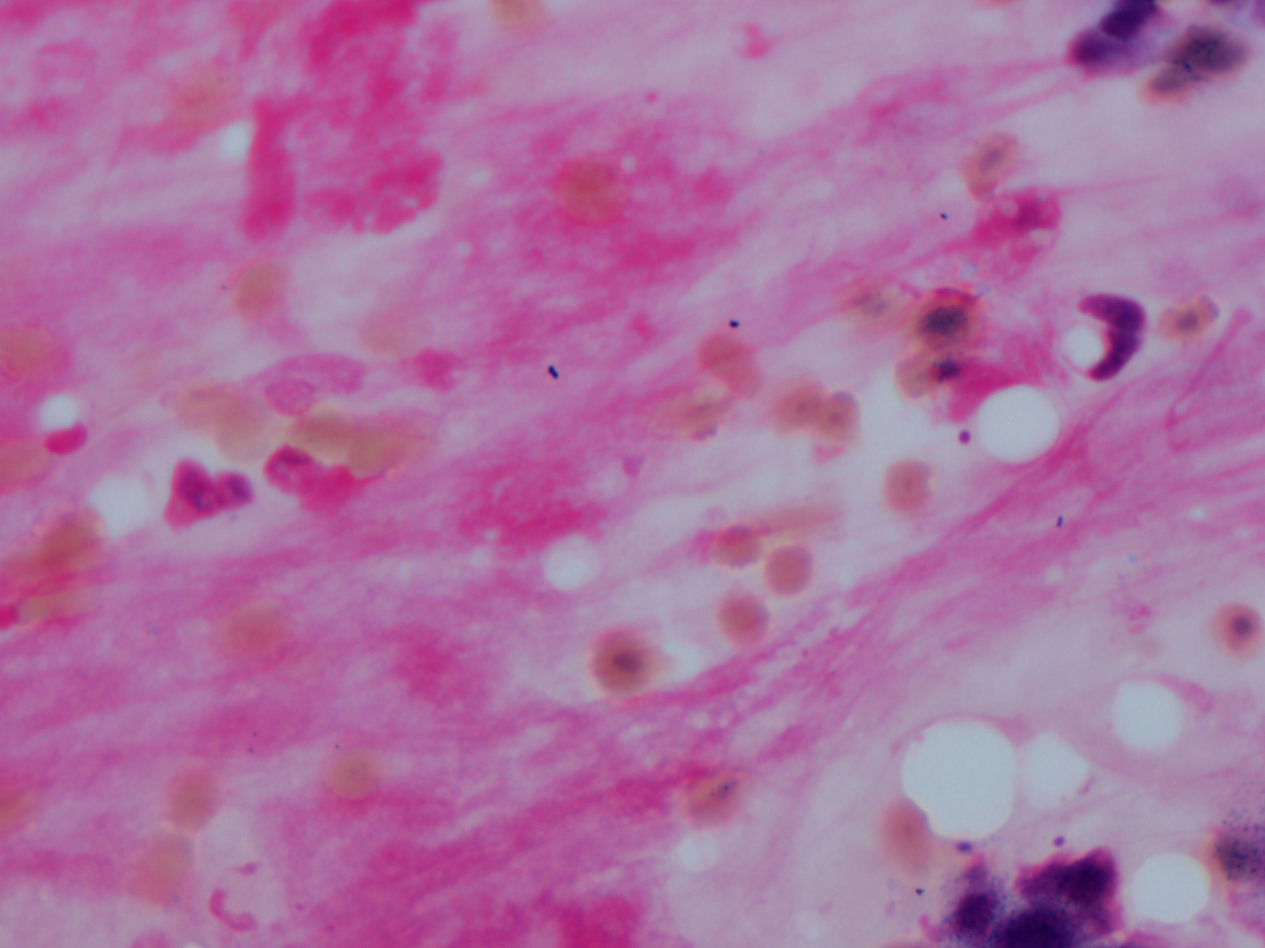
Gram-stained smear of the aspirated fluid specimen showing rare gram-positive coccobacilli and polymorphonuclear neutrophils (PMNs). Original magnification 1,000×.

Gram staining showed rare gram-positive coccobacilli with polymorphonuclear leukocytes (PMNs) (Fig. 1). The specimen was inoculated onto sheep blood agar (SBA, Condalab), chocolate agar (Oxoid), and MacConkey agar (Difco) and incubated at 37°C in a 5% CO_2_ environment. Only moderate *Staphylococcus epidermidis* growth was observed after 16–18 h of incubation.

**Fig 2:**
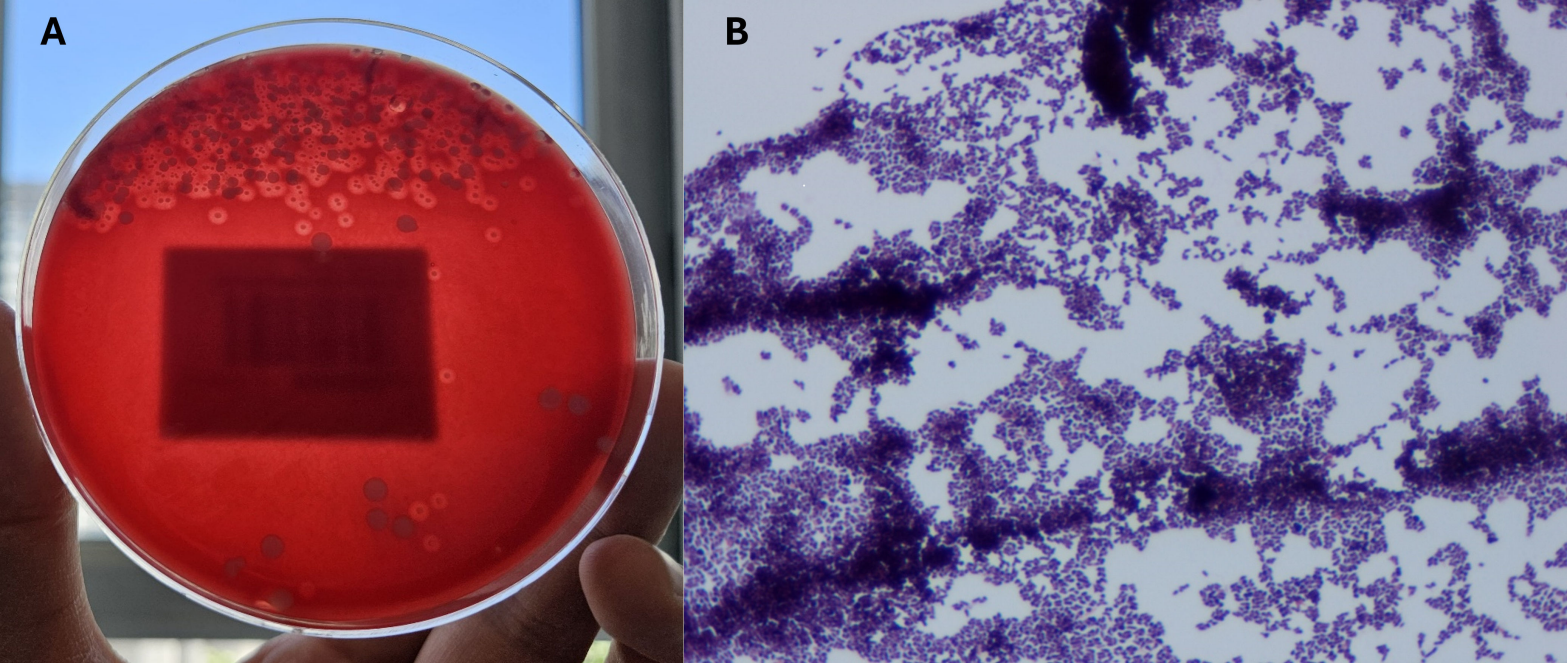
(**A**) Colony morphology and β-hemolysis zone on blood agar. (**B**) Gram-stained microscopic appearance of *Trueperella pyogenes* (original magnification 1,000×).

A moderate growth of white, opaque, shiny colonies with β-hemolysis, resembling *Streptococcus pyogenes*, appeared on SBA and chocolate agar after 42–48 h of incubation. Colonies were catalase-negative, weakly pyrrolidonyl arylamidase (PYR)-positive, and microscopically gram-positive coccobacilli (Fig. 2). Slow-growing colonies were identified as *Trueperella pyogenes* by matrix-assisted laser desorption/ionization time-of-flight mass spectrometry (MALDI-TOF MS) (Bruker Daltonics, Germany) Research Use Only (RUO) database with a 2.12 confidence score. Identification was confirmed not only by MALDI-TOF MS (RUO database) but also by biochemical characterization, including gelatin hydrolysis positivity and xylose fermentation, which supported the identification as *Trueperella pyogenes*. The combination of gelatin hydrolysis positivity and xylose fermentation was considered particularly discriminatory in differentiating *T. pyogenes* from *Arcanobacterium haemolyticum* using the API Coryne system (bioMérieux, Marcy-l'Étoile, France). As this organism is not part of the normal human flora (1), and given the patient’s animal exposure, its isolation was considered clinically significant.

Antimicrobial susceptibility testing was performed using the gradient strip test and interpreted according to the Clinical and Laboratory Standards Institute (CLSI) M45 clinical breakpoints established for *Corynebacterium* spp. and related coryneform genera. The isolate was susceptible to benzylpenicillin, cefotaxime, ceftriaxone, meropenem, vancomycin, but resistant to erythromycin, tetracycline, and trimethoprim-sulfamethoxazole (Table 1). The patient, who had a penicillin allergy, was discharged on intramuscular ceftriaxone therapy, which he had previously tolerated. During follow-up, the patient showed clinical recovery.

**TABLE 1:** Antimicrobial susceptibility of *T. pyogenes* according to CLSI and EUCAST

Antibiotics	MIC (µg/mL)	CLSI M45^*a*^	EUCAST^*b*^	
		Interpretation	Interpretation	Breakpoint (µg/mL)
Penicillin-G	0.032	S	^*c*^	0.25
Cefotaxime	0.016	S	^*c*^	0.5
Ceftriaxone	0.012	S	^*c*^	0.5
Meropenem	0.016	S	^*c*^	2
Erythromycin	>256	R	–^*d*^	–
Tetracycline	16	R	^*c*^	2
Trimethoprim-sulfamethoxazole	>32	R	^*c*^	1
Vancomycin	0.5	S	^*c*^	2

^*a*^ *Corynebacterium* spp. and related coryneform genera.

^*b*^ When there are no breakpoints in breakpoint tables? document from EUCAST.

^*c*^ The clinical use of agents for which MIC-values are higher than those listed breakpoints above should be discouraged, while agents for which the MIC is the same or lower can be considered for therapy. Avoid reporting isolates S, I, or R—instead add a comment to discourage or consider therapy.

^*d*^ –, not applicable.

## DISCUSSION

*Arcanobacterium haemolyticum*, *Trueperella bernardiae*, and *Trueperella pyogenes* are opportunistic pathogens that rarely cause infections in humans. However, infections are often clinically significant. *A. haemolyticum* is primarily associated with pharyngitis in adolescents and young adults and has also been reported in wound and soft tissue infections. *T. bernardiae* is most frequently linked to abscess formation, often in conjunction with mixed anaerobic flora (2). *T. pyogenes*, which was reclassified from the genus *Arcanobacterium* in 2011 (3), is associated with abscesses and soft tissue infections, particularly in individuals with occupational exposure to cattle, sheep, or swine. Despite the growing number of case reports, microbiological data on these organisms remain limited. It is a gram-positive, pleomorphic, non-spore-forming, non-motile, facultatively anaerobic rod (4). In the clinical microbiology laboratory, preliminary identification relies on the presence of small opaque colonies with a surrounding zone of β-hemolysis and a negative catalase reaction, which can easily be mistaken for beta-hemolytic streptococci. Microscopically, the organism appears as gram-positive coccobacilli, and it is the most important feature that distinguishes it from beta-hemolytic streptococci. *T. pyogenes* forms small, beta-hemolytic colonies after 24 h of incubation, while it forms larger colonies and beta-hemolysis after 48 h (Fig. 2). Biochemical tests are helpful in the identification of *T. pyogenes*. Catalase negativity differentiates it from *Corynebacterium spp*. (catalase positive); its non-motility distinguishes it from *Listeria monocytogenes* (motile); its ability to ferment xylose and produce gelatinase and β-glucuronidase and its negative reverse CAMP test distinguishes it from *A. haemolyticum*; and the absence of spores separates it from spore-forming bacilli. In addition, the presence of a narrow zone of β-hemolysis on blood agar and negative nitrate reduction provides further diagnostic clues. In this case, the colonies resembled *S. pyogenes*, underscoring the importance of careful evaluation of morphological and biochemical features. Importantly, *S. pyogenes*, which also shows β-hemolysis, can be distinguished by its strong PYR positivity and its sugar fermentation profile, whereas *T. pyogenes* is usually PYR-negative/weak and does not ferment sucrose or mannitol (5). Biochemical characteristics of *T. pyogenes*, *A. haemolyticum*, *T. bernardiae*, and *S. pyogenes* are shown in Table 2.

**TABLE 2:** Biochemical characteristics of *T. pyogenes*, *A. haemolyticum*, *T. bernardiae*, and *S. pyogenes^a^*

	*T. pyogenes*	*A. haemolyticum*	*T. bernardiae*	*S. pyogenes*
Gram stain	Gr(+) coccobacilli	Gr(+) coccobacilli	Gr(+) coccobacilli	Gr(+) cocci
Hemolysis, SBA	β	β	V	β
Motility	–	–	–	–
PYR	–	±w	–	+
Catalase	–	–	–	–
CAMP test	–	Rev+	–	–
Gelatin hydrolysis	+	–	–	ND
Beta-glucuronidase	+	–	–	–
Fermentation of				
Glucose	+	+	+	+
Maltose	+	+	+	+
Sucrose	+w	+w	–	+
Lactose	+	+	–	+
Mannitol	–	–	–	–
Xylose	+	–	–	ND

^*a*^ –: negative, +: positive, +w: positive (weak), Rev+: reverse CAMP positive, V: variable, and ND: no data available.

Variability in sugar fermentation tests across strains has limited the reliability of API Coryne tests for identifying *Trueperella pyogenes*. Therefore, microbiologists should be aware that although conventional biochemical kits can provide preliminary guidance, confirmatory methods such as MALDI-TOF MS or molecular assays are essential for accurate identification (5). MALDI-TOF MS has been established as an important tool for the identification of *T*. *pyogenes* and provides rapid and reliable identification with accuracy comparable to molecular techniques (6). Our findings support the existing evidence, highlighting MALDI-TOF MS as a fast and effective method for *T. pyogenes* identification (7).

*T. pyogenes* is a well-recognized opportunistic pathogen in cattle, swine, sheep, and goats and frequently causes mastitis, abscesses, and pneumonia in livestock. In contrast, human infections are rare, and reported cases are typically associated with direct or indirect contact with animals, particularly in rural settings (1, 5). Documented infections include skin and soft tissue infections, abscesses, endocarditis, prosthetic joint infections, and septicemia. In most of these cases, exposure to cattle, swine, or wildlife was reported (8–12).

Infections caused by *T. pyogenes* in humans have been frequently reported in immunocompromised individuals. Nevertheless, infections in immunocompetent individuals are increasingly recognized. For example, a case of pharyngitis was reported in a hunter following contact with a deer carcass. In another report, sepsis occurred in a previously healthy farmer (13). A prosthetic joint infection was also described in an immunocompetent patient (11, 12). These examples illustrate that zoonotic transmission is possible even in otherwise healthy hosts. Our case adds to this body of evidence by documenting soft tissue infection in an immunocompetent individual with a clear history of animal exposure through cattle slaughtering.

Antimicrobial susceptibility testing of *T. pyogenes* presents a challenge. While there are no established clinical breakpoints for this organism in the European Committee on Antimicrobial Susceptibility Testing (EUCAST), the CLSI M45 document has MIC breakpoint table for *Corynebacterium* spp. and related the coryneform genera. In this table, coryneform genera contain *Trueperella* and *Arcanobacterium* species. Although the broth microdilution method is considered the gold standard for MIC testing, standard protocol conditions, including the use of horse blood-supplemented media, could not be fully achieved; therefore, MIC values were determined by gradient strip test (*E* test) and interpreted according to CLSI M45 breakpoints. The isolate was susceptible to benzylpenicillin, cefotaxime, ceftriaxone, meropenem, and vancomycin, but resistant to erythromycin, tetracycline, and trimethoprim-sulfamethoxazole (Table 1).

For laboratories such as ours that use EUCAST standards, EUCAST provides MIC and zone diameter breakpoints for *Corynebacterium* spp.; however, there is no guidance on the use of these breakpoints for *Trueperella*. EUCAST has published a document for isolates lacking breakpoint tables (https://www.eucast.org/bacteria/clinical-breakpoints-and-interpretation/when-there-are-no-breakpoints/). It provides MIC breakpoints for gram-positive, gram-negative, and anaerobic bacteria that are not included in the breakpoint tables. When reporting susceptibility results using these breakpoints, EUCAST recommends avoiding categorical (S, I, and R) reporting and adding a comment stating that antibiotics with MIC values higher than the breakpoints should be discouraged, whereas antibiotics with MIC values that are the same or lower than the breakpoints may be considered for therapy. Therefore, beta-lactams and vancomycin were considered for therapy, and tetracycline, erythromycin, and trimethoprim-sulfamethoxazole were especially discouraged in our case (Table 1).

Antimicrobial susceptibility data of human clinical isolates are limited. Stuby et al. reported that six endocarditis cases described in the literature were susceptible to penicillin, ceftriaxone, and macrolides; however, resistance to trimethoprim-sulfamethoxazole was common, with three of four isolates resistant (9). Kavitha et al. reported that three isolates were uniformly susceptible to penicillin, ampicillin, ceftriaxone, gentamicin, and resistant to trimethoprim-sulfamethoxazole. Erythromycin and tetracycline resistance was observed in one isolate (10). Although macrolide resistance is rare among reported human cases, erythromycin resistance was reported in 22.8% of animal isolates (14). In this case, the isolate was susceptible to penicillin, ceftriaxone, and meropenem, but resistant to erythromycin, tetracycline, and trimethoprim-sulfamethoxazole.

An important clinical consideration is antibiotic selection in patients with β-lactam allergy. In this case, the patient tolerated ceftriaxone, which enabled successful treatment. However, options may be more limited in cases of true anaphylaxis since resistance to trimethoprim-sulfamethoxazole is frequent, and macrolide resistance may also be detected. This highlights the need for clear interpretive guidelines and more systematic data on human isolates to guide therapy.

From an educational perspective, this case illustrates several important lessons for clinical microbiology practice. First, *T. pyogenes* can mimic *S. pyogenes* morphologically and biochemically, and accurate identification requires awareness of this possibility. Second, MALDI-TOF MS provides a rapid and reliable means of recognition, preventing misidentification. Third, zoonotic pathogens, though rare in humans, should always be considered in patients with animal contact, even when they are immunocompetent. Finally, the absence of standardized antimicrobial breakpoints for *T. pyogenes* underscores the need for cautious interpretation of susceptibility results and highlights the value of close clinician–laboratory collaboration.

## SELF-ASSESSMENT QUESTIONS

1. In a patient presenting with a soft tissue infection and beta-hemolytic colonies resembling *Streptococcus pyogenes* on blood agar, which laboratory feature is most helpful in identifying *Trueperella pyogenes*?
  - Gram-positive coccobacilli morphology.
  - Positive PYR and bacitracin sensitivity.
  - Catalase-positive colonies with club-shaped bacilli.
  - Beta-lactamase production and oxidase-positive result.
2. In which clinical context are human infections with *T. pyogenes* most frequently reported?
  - Urban hospital outbreaks without zoonotic exposure.
  - Patients with animal contact, especially in rural areas.
  - Neonatal nosocomial infections.
  - Foodborne outbreaks in the community.
3. Which simple biochemical test most reliably distinguishes *Trueperella pyogenes* from *Corynebacterium* spp*.* in the clinical microbiology laboratory?
  - PYR test.
  - Catalase test.
  - Nitrate reduction test.
  - Sugar fermentation tests.

## SELF-ASSESSMENT ANSWERS

1. In a patient presenting with a soft tissue infection and beta-hemolytic colonies resembling *Streptococcus pyogenes* on blood agar, which laboratory feature is most helpful in identifying *Trueperella pyogenes*?
  - Gram-positive coccobacilli morphology.
  - Positive PYR and bacitracin sensitivity.
  - Catalase-positive colonies with club-shaped bacilli.
  - Beta-lactamase production and oxidase-positive result.

Answer: a. *T. pyogenes* appears as gram-positive coccobacilli and is catalase negative, with colonies that may resemble *Streptococcus pyogenes*.

1. In which clinical context are human infections with *T. pyogenes* most frequently reported?
  - Urban hospital outbreaks without zoonotic exposure.
  - Patients with animal contact, especially in rural areas.
  - Neonatal nosocomial infections.
  - Foodborne outbreaks in the community.

Answer: b. Most human infections occur in individuals with direct or indirect animal contact, particularly in rural settings.

1. Which simple biochemical test most reliably distinguishes *Trueperella pyogenes* from *Corynebacterium spp*. in the clinical microbiology laboratory?
  - PYR test.
  - Catalase test.
  - Nitrate reduction test.
  - Sugar fermentation tests.

Answer: b. The catalase test is most reliable, since *Corynebacterium* spp. are catalase positive, while *T. pyogenes* is catalase negative.

TAKE-HOME POINTS

- *Trueperella pyogenes* is a zoonotic pathogen primarily found in animals but can occasionally cause human infections.
- Infections are typically associated with animal contact but may also occur without clear exposure.
- Morphology and hemolysis patterns may mimic *Streptococcus pyogenes*, so accurate laboratory identification is crucial.
- MALDI-TOF MS provides rapid and reliable identification, reducing the risk of misclassification.
- β-lactams remain the most effective therapeutic option, although no standardized human breakpoints exist.
